# Is the Taklimakan Desert Highway Shelterbelt Sustainable to Long-Term Drip Irrigation with High Saline Groundwater?

**DOI:** 10.1371/journal.pone.0164106

**Published:** 2016-10-06

**Authors:** Jianguo Zhang, Xinwen Xu, Shengyu Li, Ying Zhao, Afeng Zhang, Tibin Zhang, Rui Jiang

**Affiliations:** 1 Ministry of Agriculture Key Laboratory of Plant Nutrition and the Agri-environment in Northwest China, Northwest A&F University, Yangling Shaanxi, China; 2 Xinjiang Institute of Ecology and Geography, Chinese Academy of Sciences, Urumqi, China; 3 Institute of Soil and Water Conversation, Northwest A&F University, Yangling, Shaanxi, China; Texas Tech University, UNITED STATES

## Abstract

Freshwater resources are scarce in desert regions. Highly saline groundwater of different salinity is being used to drip irrigate the Taklimakan Desert Highway Shelterbelt with a double-branch-pipe system controlling the irrigation cycles. In this study, to evaluate the dynamics of soil moisture and salinity under the current irrigation system, soil samples were collected to a 2-m depth in the shelterbelt planted for different years and irrigated with different groundwater salinities, and soil moisture and salinity were analyzed. The results showed that both depletion of soil moisture and increase of topsoil salinity occurred simultaneously during one irrigation cycle. Soil moisture decreased from 27.4% to 2.4% for a 15-day irrigation cycle and from 26.4% to 2.7% for a 10-day-cycle, respectively. Topsoil electrical conductivity (EC) increased from 0.64 to 3.32 dS/m and 0.70 to 3.99 dS/m for these two irrigation cycles. With increased shelterbelt age, profiled average soil moisture (0–200 cm) reduced from 12.8% (1-year) to 7.1% (10-year); however, soil moisture in 0–20-cm increased, while topsoil salinity decreased. In addition, irrigation salinity mainly affected soil salinity in the 0–20-cm range. We conclude that water supply with the double-branch-pipe is a feasible irrigation method for the Taklimakan Desert Highway Shelterbelt, and our findings provide a model for shelterbelt construction and sustainable management when using highly saline water for irrigation in analogous habitats.

## Introduction

Global climate change and population growth increase the need for water to irrigate crops for food, fiber production, and ecological sustainability [1,2]. As a result, competition for limited water supplies will increase. In arid and semi-arid regions, desertification control and shifting dune stabilization are key concerns for many people [3,4]. In addition, water resources, especially good quality water, are in short supply in most inland regions and many coastal areas [5,6] because of high evapotranspiration [7,8] and expanding cropping areas [9,10]. Thus, the safe use of marginal quality waters, such as saline water, could play a critical role in easing competition for scarce water resources in many arid and semi-arid regions [11–14]. However, poor irrigation management can lead to soil salinization, especially when using saline water for irrigation, and can also cause pollution of surface water bodies and groundwater [15]. A large number of studies have demonstrated that saline irrigation could significantly influence dynamics and distribution of soil moisture and salt, inhibit plant growth and reduce crop yield. If salts added to the soil through irrigation water are not adequately leached, the level of soil salinity will gradually increase. Thus, sustainable irrigation management strategies are very important when using saline water to reduce ecological risks.

The Taklimakan Desert is the second largest shifting desert in the world, located in the heartland of the Eurasian continent. The desert is known as the “Sea of Death” and is an extremely harsh environment with most areas being covered by shifting sand. Abundant petroleum resources were prospected in this region in the 1980s. To improve transportation for the exploitation of petroleum resources, the Taklimakan Desert Highway was completed in 1995. The highway runs north to south, has a length of 522 km, and is the longest highway across a shifting desert in the world. The highway has greatly facilitated the exploration of regional petroleum resources and the development of the economy of Southern Xinjiang. In 2003, the Taklimakan Desert Highway Shelterbelt (TDHS), a biological engineering project to protect the highway from blowing sand, was constructed on both sides along the highway. The shifting sand dunes on both sides of the highway were successfully stabilized and the regional microclimate and ecological environment were significantly improved [16].

**Fig 1:**
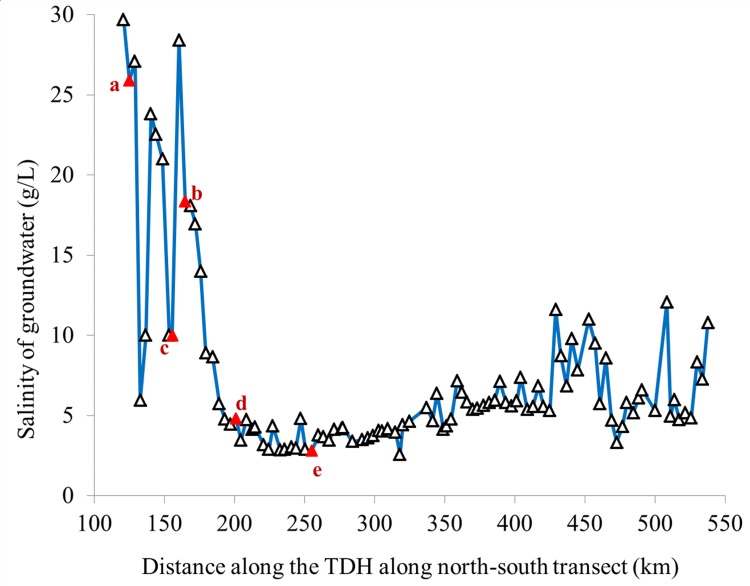
Distribution of salinity of groundwater used to irrigate the shelterbelt along the Taklimakan Desert Highway. Red triangles indicate the sites where soil samples were taken and analyzed for salinity and water content, (a) 25.90 g/L, (b) 18.39 g/l, (c) 10.00 g/L, (d) 4.82 g/L, and (e) 2.82 g/L.

Drip irrigation is one of the most efficient irrigation methods and has been widely used, especially in arid and semiarid regions where water resources are in shortage. Under saline drip irrigation, soluble salts along with water can be pushed toward the fringes of wetting area, and a desalinization zone forms in the center of wetting area near the dripper [17,18]. If reasonably managed, the soil salinity would not increase with long term saline drip irrigation [18–20]. Thus, saline water is widely used to drip irrigate crops [21]. Xu et al. [22] showed that drip irrigation was the optimal method for low-cost and water-saving irrigation for shelterbelt construction in the Taklimakan Desert. All plants of the TDHS are drip-irrigated with high-salinity (2.82–29.70 g/L, Fig 1) groundwater because of freshwater shortage [13]. Irrigation water salinity strongly affects the survival and growth of the shelterbelt plants [23]. Soil salinity also decreases soil organic carbon stocks [24]. Thus, whether long-term drip irrigation with highly saline groundwater would result in soil salinization, and whether local saline groundwater can be safely applied for irrigation in this region, are important concerns.

Therefore, to prevent soil salinization and ensure sustainability of the shelterbelt, we examined the irrigation management practices for a period of more than 10 years along the TDHS and in the hinterland of the Taklimakan Desert. The objective of this study was to evaluate dynamics of soil moisture and salt under current irrigation systems, provide theoretical basis for sustainable management of the shelterbelt, and to provide insights for addressing problems in analogous habitats elsewhere in the world.

## Materials and Methods

### The study area

The study was carried out at the Taklimakan Desert Research Station (TDRS) and along the Taklimakan Desert Highway (TDH) in the Xinjiang Autonomous Region, China. The station is located in the heartland of the Taklimakan Desert (38°58’ N, 83°39’ E, 1100 m.a.s.l.). Most of the area along the highway and near the station is covered with shifting sand, and natural vegetation is very sparse except for a few drought- and salt-resistant shrubs growing in inter-dune areas (e.g. *Tamarix taklamakanensis* M.T. Liu and *Calligonum taklimakanensis* B.R. Pan & G.M. Shen). The landscapes are mainly high-shifting dunes and large complex dune chains. The soil is mainly shifting Aeolian Sandy Soil with a very low nutrient content [25], a salt content of 1.26–1.63 g/kg, and a pH of 8–9 (Table 1). The groundwater salinity used to irrigate the shelterbelt near the station is approximately 4.04 g/L. The groundwater levels at the inter-dunes are relatively shallow, with a depth of 3–5 m. The ions in the groundwater are mainly Cl^-^, SO_4_^2-^, Na^+^, and K^+^. The water quality profiles of selected experimental sites along the TDH are shown in Table 2.

**Table 1:** Physicochemical properties of shifting Aeolian Sandy Soil at the study area.

pH (1:2.5)	Total salt content (g/kg)	Ionic composition (g/kg)								Particle composition (%)		
		CO_3_^2-^	HCO_3_^-^	Cl^-^	SO_4_^2-^	Ca^2+^	Mg^2+^	Na^+^	K^+^	Clay	Silt	Sand
8.3	1.33	0.02	0.106	0.703	0.014	0.096	0.01	0.32	0.061	0.27	12.35	87.38

**Table 2:** Profiles of irrigation water quality of selected sites.

Serial number	Geographical coordinate	Location along TDH (km)	Salinity (g/L)	pH	Ionic composition (g/L)					
					HCO_3_^-^	Cl^-^	SO_4_^2-^	Ca^2+^	Mg^2+^	K^+^ & Na^+^
a	40°46’ N, 84°18’ E	125.00	25.90	7.69	0.21	10.57	5.87	0.72	0.92	7.41
b	40°25’ N, 84°19’ E	164.80	18.36	8.09	0.18	6.54	5.07	0.40	0.61	5.14
c	40°29’ N, 84°19’ E	155.80	10.00	8.02	0.09	4.37	2.14	0.24	0.31	2.84
d	40°05’ N, 84°19’ E	201.10	4.82	7.85	0.07	1.80	1.07	0.10	0.25	1.52
e	39°39’ N, 84°05’ E	255.20	2.82	7.40	0.06	1.07	0.69	0.07	0.09	0.83
f	38°58’ N, 83°39’ E	TDRS	4.04	7.76	0.08	1.50	1.01	0.11	0.15	1.07

TDRS = the Taklimakan Desert Research Station. 0 km is the northerly starting point of the desert highway.

The Taklimakan Desert Highway Shelterbelt (TDHS) has a length of 436 km, a width of 72–78 m, and an area of 3 128 km^2^. It is oriented in north to south direction across the desert. Between locations Luntai and Xiaotang is an 86-km section with no artificial shelterbelt because of relatively good natural vegetation on both sides of the TDH [23,26]. Plants grown in the shelterbelt are mainly drought- and salt-tolerant species with excellent windbreak and sand fixation properties, including *Calligonum arborescens* Litv., *Tamarix ramosissima* Ledeb., and *Haloxylon ammodendron* (C. A. Mey.) Bunge. The plants were interspersed along rows, with a row spacing of 2 m and a plant spacing in the same row of 1 m. Plants are drip-irrigated with highly saline groundwater (salinity: 2.82–29.87 g/L) pumped from local wells [13,22]. On both sides of the highway the topography is heterogeneous and complex [27]. As a result of this topography, the types of wind-blown sand events vary significantly along the highway and require the use of different shelterbelt structures to control wind-blown sand [28]. Because there are great differences among the salt tolerance of plants and groundwater salinities, species combinations at different sections of the shelterbelt differ. Before the shelterbelt construction, several species with a high stress-resistance were selected from more than 200 species after more than 10 years’ research on plant introduction [29]. One prototype section of a shelterbelt covering 7 km was established to assess the feasibility of shelterbelt construction along the highway. The shelterbelt along the TDH was constructed in 2003, and the shelterbelts near the TDRS were constructed in stages and vary in age from 1 to 11 years. The height, crown width and maximum width of basal stem of the plants increased with the shelterbelt age, and have different morphology [23].

We state that no specific permissions were required for these locations/activities. We conform that the field studies did not involve endangered or protected species.

### Irrigation systems

**Fig 2:**
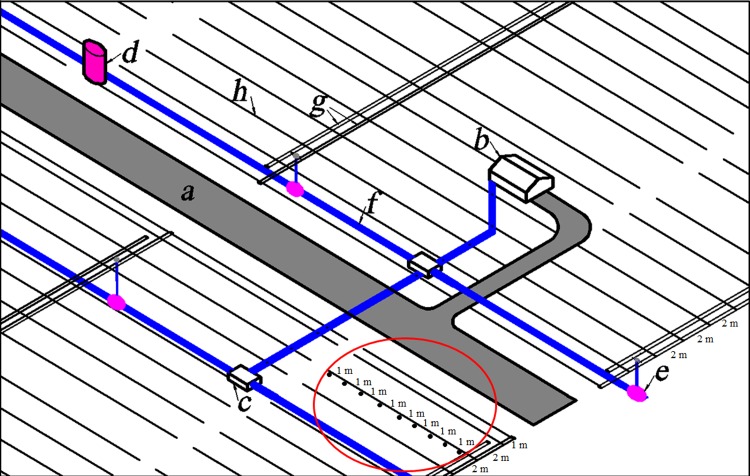
Schematic diagram of the drip irrigation system for the Taklimakan Desert Highway Shelterbelt. (a) Desert highway; (b) Well-house; (c) Control valve well; (d) Connecting well; (e) Water valve; (f) Main pipe; (g) Double-branch pipe; (h) Capillary tube. The spacing between capillary tubes is 2 m or 1 m. The spacing of the two adjacent emitters along the same capillary tube (black dots shown in the red circle) is 1 m, and all the capillary tubes are the same size.

The irrigation systems used in the TDHS and the TDRS (Fig 2) are mainly drip irrigation, which was shown to be the best irrigation method [22]. After groundwater being pumped from the well (Fig 2B), it is imported to water valves (Fig 2G) through main pipes (Fig 2F) and control valve wells (Fig 2C). Water valves connect to a double-branch pipe and control the irrigation speed and irrigation time. The double-branch-pipe was designed for effectively supplying water for different plants and was used for the shelterbelt irrigation. *Calligonum arborescens*, *Tamarix ramosissima*, and *Haloxylon ammodendron* were interspersed along rows. The root distribution and water consumption of these plants are different [30,31]. water consumption is most pronounced for *C*. *arborescens* because it has shallower roots than the other two plants [30,32], and moisture depletion of then shallow soil is faster because of plant uptake and atmospheric evaporation, and therefore it must be irrigated more frequently than the other two plants. The irrigation frequency of *C*. *arborescens* is set at 10 days, and *T*. *ramosissima* and *H*. *ammodendron* are set at 15 days. Fertilizer tanks are connected with irrigation systems for the application of soluble fertilizers to improve plant growth. Fertilizers are commonly used at the early stage of the growth season or when the plants grow weak.

### Climate data collection

Climate data were collected from the automatic weather station located at the TDRS for more than 10 years (2003–2014). This weather station is the only one along the desert highway. The data include atmospheric relative humidity, precipitation, evaporation, temperature, and wind speed and direction. The data were automatically recorded daily, and average monthly values for these parameters were calculated.

### Soil sampling and analysis

Shelterbelts in the TDRS had been planted for 1, 4, 7, and 10 years and are drip irrigated with a salinity of 4.04 g/L, because this salinity is most common and is used to irrigate the largest area near the TDRS. In July 2014, monitoring took place adjacent to sets of five drip-emitters. Soil samples 30 cm from the emitters were collected daily before and after irrigation for a 15-day irrigation period for *T*. *ramosissima* and *H*. *ammodendron*, and a 10-day irrigation period for *C*. *arborescens*, respectively. All samples were collected to a 200-cm depth at 9:00 am at 20-cm increments. Soil moisture-salinity dynamics in one irrigation cycle were averaged for four shelterbelt ages, and moisture-salinity distribution for each differently aged shelterbelt was averaged from daily data.

In addition, for a shelterbelt planted in 2003, sets of five emitters, each irrigated with groundwater having salinities of 2.82, 4.82, 10.00, 18.36, and 25.90 g/L (Fig 1), were monitored to a depth of 200 cm with increments of 20 cm during one irrigation cycle. Sampling plots were 30 cm from the emitters and perpendicular to the plant rows.

All samples were divided into two portions for moisture analysis and electrical conductivity (*EC*) measurement. Soil moisture was determined by mass loss after drying at 105°C for 12 h. The daily soil water storage (*W*_*c*_) in one irrigation cycle was calculated by [33]:

$$W_{c}=V_{s}\;*\;H$$

where *W*_*c*_ (mm) is soil moisture storage; *V*_*s*_ (cm^3^/cm^3^) is soil water content; and *H* (mm) is the thickness of the soil layer.

Another portion of the samples was air-dried and sieved through a 1-mm sieve. Soluble salt estimates were then based on extracts of one part soil to five parts water by weight, and the *EC* was determined using a conductivity meter. All values were averaged from five duplicates.

Data for the 1, 4, 7, and 10-year shelterbelts irrigated with 4.04 g/L salinity groundwater were used to analyze the daily dynamics of soil moisture and salinity. Average values for the shelterbelts planted for different years were used to analyze the effect of shelterbelt age on soil moisture-salinity distribution. Average values for shelterbelts irrigated with different salinity were used to analyze the effect of irrigation salinity on soil moisture and salinity distribution.

## Results

### Climate

**Fig 3:**
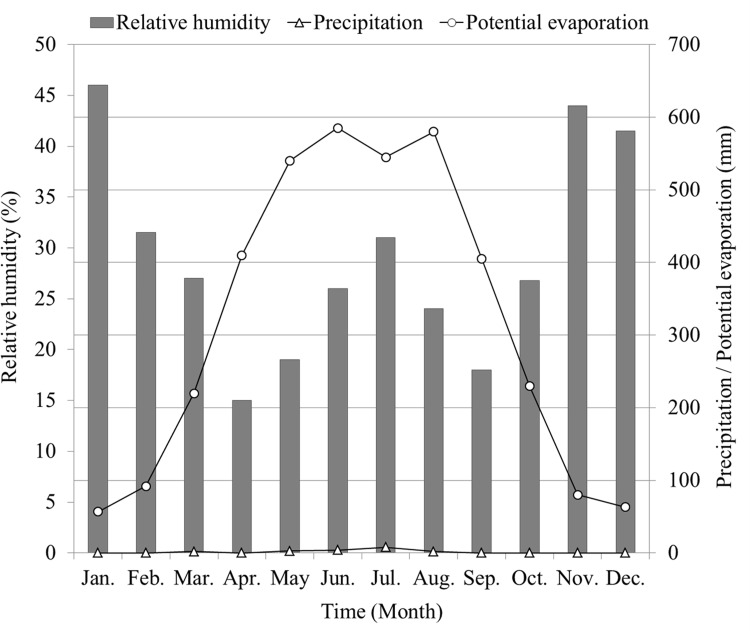
Relative humidity, precipitation, and potential evaporation in the hinterland of the Taklimakan Desert.

The irrigation schedule is based on the regional climate. The annual mean atmospheric temperature of the study area is 12.4°C, with the coldest month of −8.1°C in December, and the warmest month of 28.2°C in July. The highest and lowest recorded atmospheric temperatures are 43.2°C and −19.3°C, respectively. The area is hyper-arid with an average annual precipitation of 24.6 mm (Fig 3). With a mean potential evaporation (*E*_*p*_) of up to 3639 mm through the year, precipitation can virtually be ignored when compared with *Ep*. The average relative humidity (*RH*) is only 29.4%, and 247 days are of low-humidity (≤ 30%).

We discovered that wind-blown sand storms are serious and the average wind speed exceeds 2.5 m/s, maximum instantaneous wind speed is up to 20.0 m/s, and the sand-moving winds occur annually for more than 130 days. From Table 3, it can be seen that winds mainly occur from April to August, and the direction is mainly ENE, and sand-moving winds (≥ 6.0 m/s) blow for 550–800 h/yr. Winter (from November to the following February) has a low evaporation because of the low temperatures, higher *RH*, fewer hours of wind, and low-speed winds. Though the temperature is highest in July, potential evaporation is not at its maximum because of a relatively high *RH*. The climate of the study area can be summarized as extremely arid with very limited precipitation and strong evaporation demand, high atmospheric temperature in summer, and large periods of winds with high speeds.

**Table 3:** Monthly number of sand-moving winds with a speed of ≥ 6 m/s (average number for 2003–2014).

Month	Number of sand-moving winds																Sum
	N	NNE	NE	ENE	E	ESE	SE	SSE	S	SSW	SW	WSW	W	WNW	NW	NNW	
Jan.	—	—	—	—	1	—	—	—	—	—	—	—	—	—	—	—	1
Feb.	—	—	—	—	1	—	—	—	—	—	—	—	—	—	—	—	1
Mar.	1	1	8	9	—	1	1	—	—	—	1	1	—	—	—	—	23
Apr.	—	2	13	45	19	5	—	—	—	—	—	—	—	—	—	—	**84**
May	—	—	5	18	8	3	—	—	—	5	5	7	8	3	2	2	**67**
Jun.	13	17	9	13	6	2	—	1	2	4	1	4	8	2	4	4	**90**
Jul.	3	15	16	15	8	4	—	1	3	2	—	—	—	1	2	1	**71**
Aug.	9	10	19	23	13	3	2	2	1	2	3	9	6	3	—	1	**106**
Sep.	—	—	4	10	12	4	—	—	—	—	—	—	—	—	—	—	30
Oct.	—	—	2	—	—	—	—	—	—	—	—	—	—	—	—	—	2
Nov.	1	—	6	1	2	—	—	—	—	—	2	—	—	—	—	1	13
Dec.	—	1	—	—	1	—	—	—	—	—	—	—	—	—	—	—	2
Sum	27	46	**82**	**134**	**71**	22	3	4	6	13	12	21	22	9	8	9	

—: indicates that there was no sand-moving wind.

### Soil moisture and salinity

#### Soil moisture-salinity dynamics for one irrigation cycle

**Fig 4:**
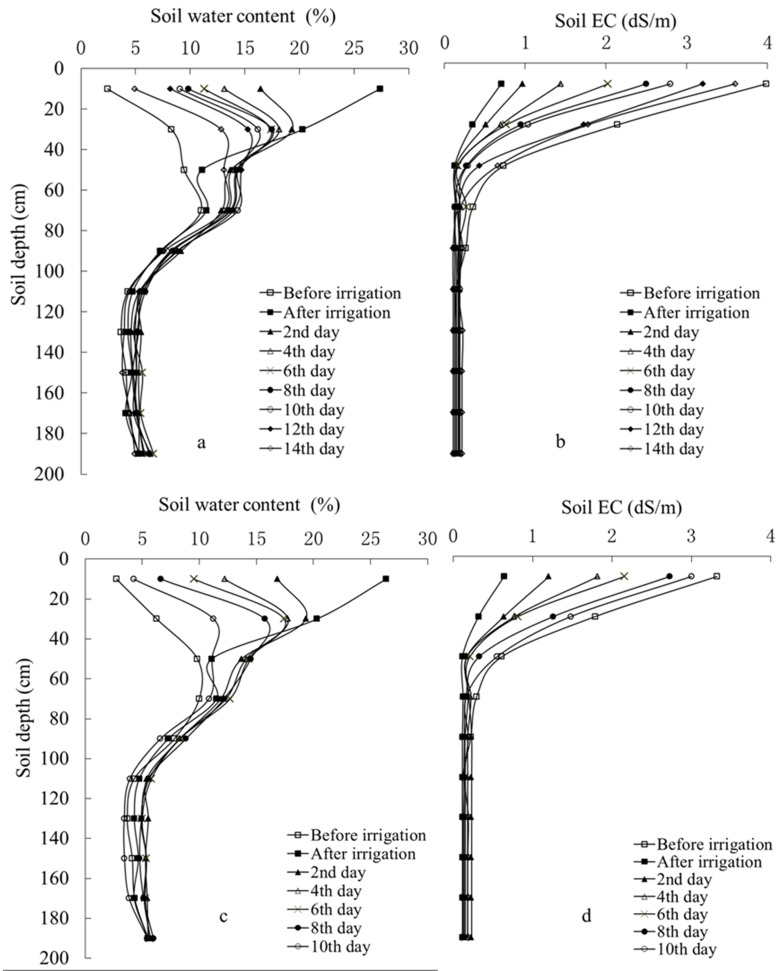
Dynamics of soil moisture and salinity within an irrigation cycle. (a,b) Soil moisture and salinity of 15-day-cycle for *Tamarix ramosissima* and *Haloxylon ammodendron*; (c,d) Soil moisture and salinity of 10-day-cycle for *Calligonum arborescens*.

The moisture dynamics of the 2-m soil profile (Fig 4A and 4C) varied significantly, especially for the depth of 0–80 cm where the moisture is easily affected by evaporation, infiltration, and root uptake. Water applied to sandy soil infiltrates fast, so soil moisture at 0–40 cm rises quickly during irrigation. Evaporation and infiltration of soil water occur simultaneously during and after irrigation, and infiltration eventually ceases by the 2nd day after irrigation. Soil moisture decreased fast at 0–40 cm after irrigation, decreased slower at 40–80 cm and was relatively stable beneath 80 cm. In addition to soil moisture, the salinity of the 0–50-cm soil zone (Fig 4B and 4D) varied significantly for different days after irrigation. Especially for the surface soil, EC increased from 0.7 to 3.6 dS/m for 15-day-cycle and 0.6 to 3.0 dS/m for 10-day-cycle, respectively. But deeper soil shows effectively no variation with a value lower than 1 dS/m.

**Fig 5:**
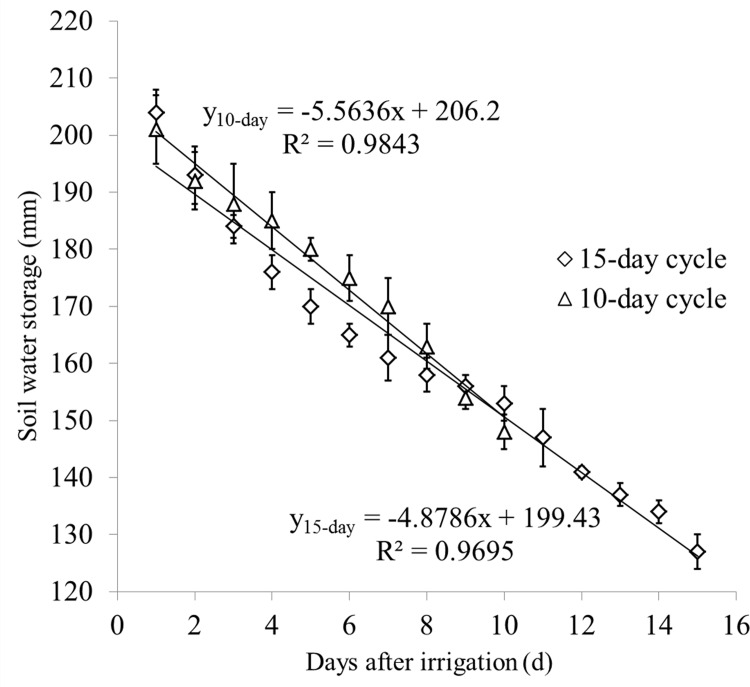
Dynamics of soil water storage to a depth of 2 m within an irrigation cycle. a: 15-day-cycle for *Tamarix ramosissima* and *Haloxylon ammodendron*; b: 10-day-cycle for *Calligonum arborescens*.

Fig 5 showed that soil water storage (*W*_*c*_) at 0–200 cm depth decreased after irrigation. *W*_*c*_ was 125 mm (15-day) and 127 mm (10-day) before irrigation and increased to 204 mm (15-day) 201 mm (10-day) immediately after irrigation. Thereafter, soil water storage decreased 79 mm and 74 mm during one irrigation cycle. The relationship between *W*_*c*_ and the days after irrigation showed a clear linear relationship, as shown in Fig 5.

#### Soil moisture-salinity depth profiles in shelterbelts of different age

**Fig 6:**
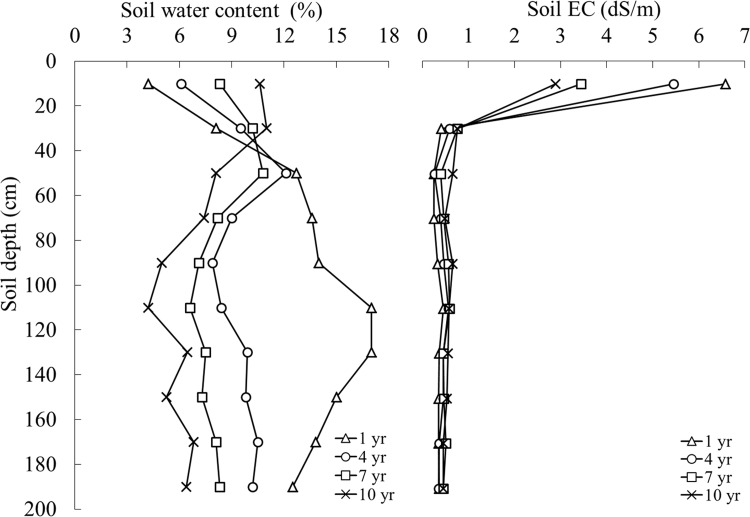
Depth profiles of soil moisture and salinity in shelterbelts of different age.

Fig 6 shows the variation of moisture and salinity for shelterbelts planted for 1, 4, 7, and 10 years. The moisture decreased significantly with increasing shelterbelt age (Fig 6-Left). Average moisture was 12.8% for the 1-year-old shelterbelt and decreased to 9.3%, 8.2%, and 7.1% for 4-, 7-, and 10-year-old shelterbelts, respectively.

The soil EC at a depth of 0–20 cm significantly reduced with shelterbelt age (Fig 6-Right). Topsoil EC under shelterbelts planted for 1, 4, 7, and 10 years were 6.58, 5.45, 3.44, and 2.89 dS/m, respectively. However, soil EC at depths below 20 cm was not affected by irrigation and showed little variation.

#### Soil moisture-salinity depth profiles when irrigated with different salinity groundwater

**Fig 7:**
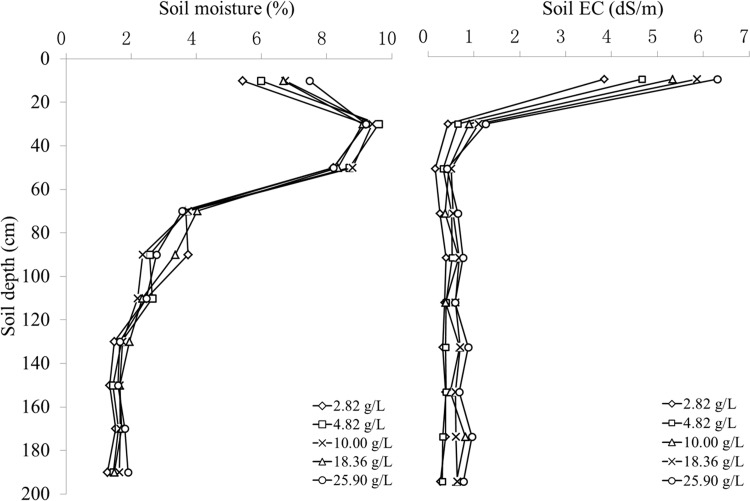
Depth-distribution of soil moisture and salinity at 0–2-m depth irrigated with different salinity groundwater.

Fig 7 shows how the distribution of soil moisture and salinity varied when drip-irrigated with different salinity groundwater. With the same irrigation amount and cycle, soil moisture at different depth and irrigated with different salinity groundwater showed a depth profile (Fig 7-Left). We deduced that water consumption of shelterbelt plants is not affected by salinity. Irrigation water salinity significantly influenced shallow soil salinity and clearly increased with water salinity (Fig 7-Right). The highest and lowest salinity of surface 20 cm soil were 6.31 dS/m and 3.84 dS/m when irrigated with 25.90 g/L (highest) and 2.82 g/L (lowest) salinity water, respectively. However, this salinity relationship did not occur in soil layers deeper than 20 cm.

## Discussion

Because of sparse precipitation and a lack of available freshwater in the Taklimakan Desert, groundwater is the only irrigation water source for the shelterbelt plants, soil moisture and salinity are two most important factors which control plant growth. The variation of soil moisture can be influenced by the irrigation amount, root distribution, soil physical properties, and climate [21,33]. Hyper-arid climate inevitably causes high water consumption. According to water consumption of the shelterbelt plants and soil moisture evaporation in the shelterbelt around the year related to local climate [31,34], a specially designed irrigation systems using double-branch-pipe was implemented. The depletion of the soil water content was mainly caused by soil surface evaporation and plant transpiration. The deep percolation of soil moisture is negligible because of the limited irrigation amounts. Plant growth will be significantly reduced and some plants could die if the soil moisture is too low or soil salt content of the root zone is too high [35, 36]. We examined the daily dynamics of soil moisture and salinity during one irrigation cycle in June when there is relatively high air temperature, low RH, and high evaporative demand. The soil moisture-salinity dynamics are similar during other months, while the evaporative demands may be similar or much lower. Therefore more moisture will be stored in the soil and less salt will accumulate at the soil surface, thus supporting the normal growth of shelterbelt plants.

Soil moisture at 0–40 cm decreased after irrigation, which is mainly due to the effects of high atmospheric evaporation caused by the hyper-arid climate [33]. Soil moisture at 40–80 cm is mainly affected by the root water uptake because it is the main distribution layer of the shelterbelt plant root [32], and because the atmospheric conditions have a lesser affect on this layer. Soil moisture beneath 80 cm was relatively stable because little plant roots reached that depth. Soluble salt moves upwards with moisture evaporation and causes the EC of shallow soil to increase after irrigation. Because salt resides mainly in shallow soil layers where there is little rooting, plants will not suffer salt-injury and can grow well [32].

Moisture in the 0–40-cm layer increased with shelterbelt age because of an increase in organic matter and dust accumulation [21]. The addition of organic matter and dust would therefore improve soil moisture holding capacity. Moreover, the crown width of shelterbelt plants increased substantially annually after planting and consequently reduced soil evaporation, thus increasing the shallow soil moisture in the shelterbelt [23, 37]. The results indicated that the depth of soil moisture consumption increased with the shelterbelt age because of increasing depth and amount of plant root distribution, especially moisture below 40 cm decreased [30, 32]. Soil moisture at a depth of 20–80 cm is mainly used by plants because this layer contains the majority of roots of shelterbelt plants [30, 32]. Zhang et al. found that most soluble salts accumulated at the soil surface when high-salinity irrigation water was used [38]. The results from our experiments confirm this.

Salt accumulation and leaching occur alternately at the study area under irrigation conditions. Salt accumulates in the topsoil when moisture evaporates and is affected by surface cover [39]. Salt accumulation is less as moisture evaporation is reduced with increasing shade as a result of shelterbelt plant growth [37]. Salt leaching occurs during precipitation and irrigation [38], but if there is less than 50 mm precipitation at the study area, the leaching can be ignored. One large amount of irrigation (twice normal irrigation) is essential every October to ensure salt leaching before irrigation is stopped from November to the following February. Irrigation is the main agent for salt leaching, and it appears that a larger quantity of salts is removed from the soil solution than is accumulated [40]. In addition, an irrigation tube buried by accumulated shifting sand in the shelterbelt may reduce moisture evaporation and inhibit salt accumulation [41, 42]. There is a concern that long-term saline irrigation may result in secondary soil salinization in such extreme environments [16]. However, our results indicate that several factors lead to a salt content decrease with shelterbelt age, thus making conditions more favorable for plant growth.

Water supply with a single-branch-pipe is very common for irrigation systems of crops or homogeneous plantations because of their similar demands for water consumption and relatively healthy environments for plant growth. However, in the study area, the shallow root distribution of *C*. *arborescens* combined with high evaporative demand and poor soil water retention makes it difficult for the normal growth under the 15-day irrigation cycle [30, 32], so it is irrigated with a 10-day cycle. The study results of Xu et al. [17] showed that the optimal irrigation amount for the study area was 30 L per plant per irrigation event. This irrigation amount is typically applied along the TDHS. All shelterbelt plants grow well under such irrigation systems.

In practice, the same irrigation amounts and frequencies are applied to all shelterbelts, but the variation in water consumption with shelterbelt age is not considered. Larger plants will consume more water by transpiration and cause a decrease in soil moisture. Thus, experiments on the responses of shelterbelt plants of different age (or size) to irrigation intervals and irrigation amounts should be conducted for appropriately adjusting the irrigation schedule to improve water use efficiency. Long-term field monitoring systems for soil moisture and salinity under drip irrigation with different groundwater salinities have been established along the TDHS. Based on the results of this monitoring, a prediction model for soil moisture and salinity will be developed for taking further measures to reduce, or even avoid, the risk of soil salinization. This concern could be addressed by adjusting the irrigation cycle and irrigation amounts for different shelterbelt age on the basis of experiments. For example, it is possible that irrigation amounts could be reduced for a 1-year-old shelterbelt, and increased for shelterbelt older than 10 years, leading to improved plant growth and improved water use efficiency.

The results of this study raise questions about the utility of saline groundwater drip irrigation for the TDHS with an Aeolian Sandy Soil context in the Tarim Basin of northwestern China. Irrigating with groundwater in this extreme environment may lower the groundwater level and lead to ecological degradation of the area. Fan et al. reported that the groundwater table was essentially stable during a 4-year period because irrigation accounted for less than 3% of groundwater recharge in the Tarim Basin [43].

An interesting issue is how to transfer the application of this model to analogous habitats elsewhere. This question will be answered in three steps. First, available local water resources, plant species, soil and water properties, and climatic characteristics should be generally investigated for planning purposes. Second, preliminary experiments of plant growth under saline irrigation should be conducted to evaluate plant growth and choose the most suitable plant species. Obviously, the plant root distribution and water consumption characteristics should be understood. Finally, the feasibility of large-scale implementation must be fully proven. This includes the ecological benefits and risks, social benefits, and economic costs and benefits.

## Conclusion

The study area is characterized as hyper-arid, with a very low precipitation, very low relative humidity, high potential evaporation, and frequent wind-blown sand storms. Our results showed that high-saline groundwater is a feasible source to use for irrigating artificial shelterbelts in such extremely arid desert regions. Soil moisture depletion and shallow soil salinity accumulation occurred simultaneously during one irrigation cycle. The moisture increased and salinity decreased significantly at 0–20-cm depth, but moisture below a depth of 20 cm significantly decreased with the increasing shelterbelt age, and salinity below a depth of 20 cm showed marginal variation. In addition, irrigation salinity only affected soil salinity at 0–20 cm, but had almost no effect on soil moisture and salinity deeper than 20 cm.

In summary, long-term high-salinity drip irrigation with sensible management practices will not negatively impact plant growth in the Taklimakan Desert. Our findings are helpful for ensuring the sustainability of the TDHS, and provide a model for shelterbelt construction using high-salinity water for irrigation in arid and semi-arid deserts.

## Supporting Information

S1 File Supporting information about data. (XLSX)

S2 File Supporting information about Fig 2. (DOC)
