# Supplementary figures and images for: Is the Taklimakan Desert Highway Shelterbelt Sustainable to Long-Term Drip Irrigation with High Saline Groundwater?
Source: PLoS One. 2016 Oct 6;11(10):e0164106. doi: 10.1371/journal.pone.0164106 (PMC5053434; doi:10.1371/journal.pone.0164106)

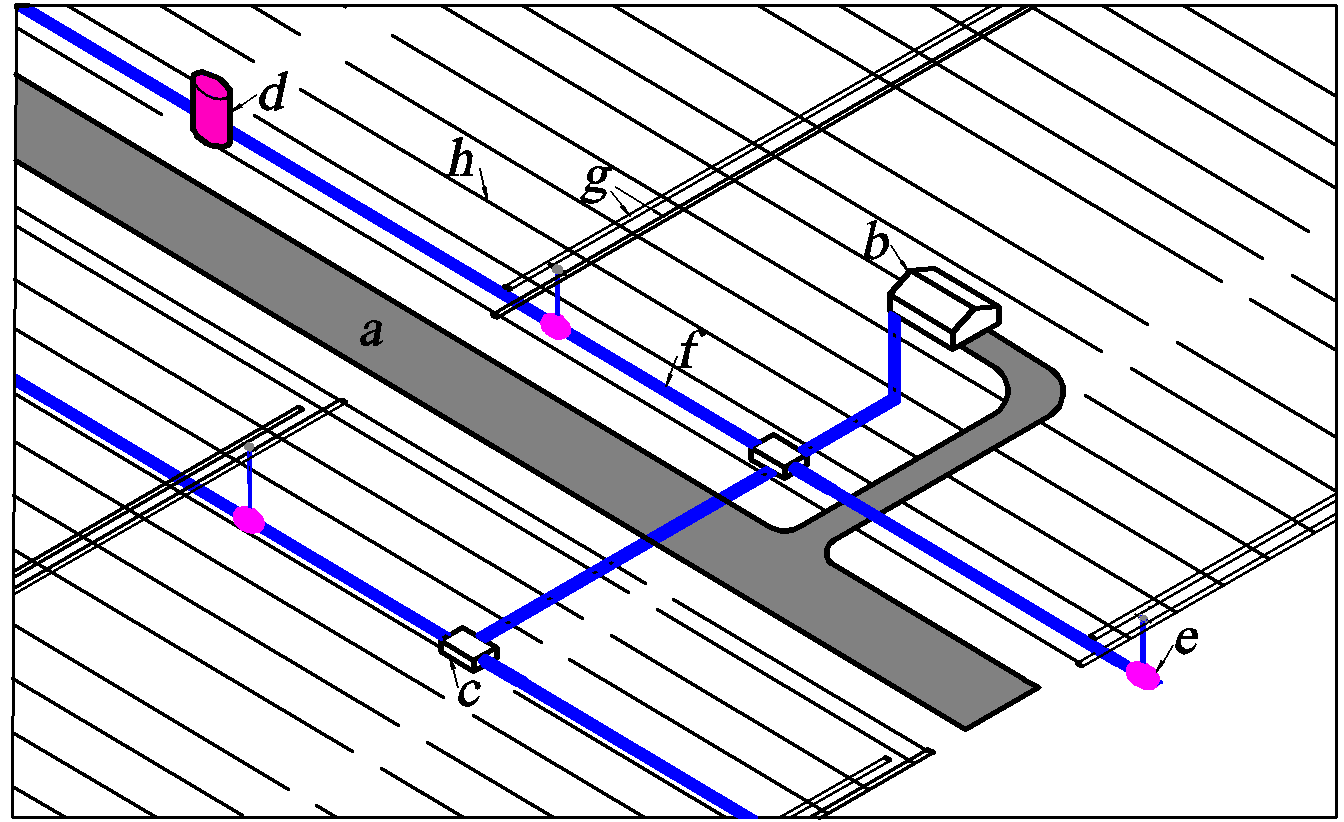


1 m

2 m

2 m

2 m

2 m

2 m

1 m

1 m

1 m

1 m

1 m

1 m

1 m

1 m

Supplement: S2 File — (DOC) [file pone.0164106.s002.doc]
